# Supplementary material for: Knowledge, attitudes and practices of healthcare workers towards noma in Zambezia, Mozambique
Source: PLoS Negl Trop Dis. 2025 Mar 19;19(3):e0012939. doi: 10.1371/journal.pntd.0012939 (PMC11952756; doi:10.1371/journal.pntd.0012939)
Supplement: S1 File — (PDF) [file pntd.0012939.s001.pdf]

KNOWLEDGE OF NEGLECTED DISEASES QUESTIONNAIRE

	**Information filled in by the researcher:**
1	Date (day - month - year): |_|_| - |_|_| - |_|_|_|
2	Health Unit:
3	Name of researcher:
4	Participant code (province code / district code / no.): |_|_| / |_|_| - |_|_|
	Geolocalisation: ____________ , ___________________
	**Participant information**
5	Sex: ☐ Male ☐ Female
6	Type of health professional (category):
7	Level of education attained (choose one):<br>☐ Primary education ☐ University education<br>☐ Secondary Education ☐ Post-University Education<br>☐ Pre-University Education
8	How many children up to the age of 5 do you see every **day**? (choose one):<br>☐ < 5 ☐ I'm not sure<br>☐ 5 - 10<br>☐ 11 - 15<br>☐ 16 - 24<br>☐ > 24
9	How many children up to the age of 5 seek care for oral health problems in a **month** (approximately)? (choose one):<br>☐ < 25 ☐ 75 - 99<br>☐ 26 - 50 ☐ > 100<br>☐ 51 - 75 ☐ I'm not sure

10	How many of these children (approximately) had bleeding and/or swollen and/or ulcerated gums? Number of children =|_|_|_| ☐ Not sure
11	How many of these children (approximately) had cheek oedema?<br>Number of children = |_|_| □ Not sure
12	How many of these children (approximately) had facial tissue necrosis?<br>Number of children = |_|_| □ Not sure
13	If a child suffers from one of the following diseases, do you examine their mouth?<br>Rubela: □ Yes ☐ No ☐ I'm not sure<br>Malaria: ☐ Yes ☐ No ☐ Not sure<br>Malnutrition: ☐ Yes ☐ No ☐ Not sure<br>HIV: ☐ Yes ☐ No ☐ Not sure

ATTITUDES AND PRACTICES: CASE STUDIES

CASE 1	A mother is looking for care for her 2-year-old son who has bleeding gums and bad breath.	MAXIMUM TOTAL POINTS:<br>**3 marks**
14	What could be the possible diagnosis?	**0.75 marks** are given for any of these diagnoses:<br>• Noma stage I<br>• Acute Necrotising Ulcerative Gingivitis<br>• Ulcerative gingivitis<br>• Necrotising gingivitis

		Just mentioning “gingivitis” or “simple gingivitis” scores 0
15	What treatment would you give?	**Treatment and advice can score a maximum of 2.25 marks**. For every of the following items, 0.25 marks where given<br>• Amoxicillin or ampicillin = 0.25<br>• Metronidazole = 0.25<br>• Ibuprofen or paracetamol or analgesics = 0.25<br>• Scaling or debridement = 0.25<br>• Nutritional support, including references to specific measures e.g. multivitamins = 0.25
16	What advice would you give?	• Nutritional support, including references to specific measures e.g. multivitamins = 0.25 (only if not mentioned in treatment already)<br>• Improve oral hygiene = 0.25<br>• Gargle with salt water = 0.25<br>• Adhere to treatment = 0.25<br>• Avoid hot and/ or spicy foods = 0.25

CASE 2	He is a 4-year-old patient with a fever and swollen lips and cheeks	MAXIMUM TOTAL POINTS:<br>**3 marks**
17	What could be the possible diagnosis?	**0.75 marks** are given for any of these diagnoses:<br>• Noma<br>• Noma stage II

		• Noma oedema stage
18	What treatment would you give?	**Treatment and advice can score a maximum of 2.25 marks**. For every of the following items, 0.25 marks where given, except for referral which scored double given its importance:<br>• Intravenous or oral amoxicillin or ampicillin or penicillin = 0.25<br>• Intravenous or oral metronidazole = 0.25<br>• Intravenous or oral gentamycin = 0.25<br>• Ibuprofen or paracetamol or analgesics = 0.25<br>• Rehydration = 0.25<br>• Nutritional support, including references to specific measures e.g. multivitamins = 0.25<br>• Referral = 0.5
19	What advice would you give?	• Nutritional support, including references to specific measures e.g. multivitamins = 0.25 (only if not mentioned in treatment already)<br>• Adhere to treatment = 0.25

CASE 3	We have a 25-year-old patient with gangrenous tissue and a hole in her cheek:	MAXIMUM TOTAL POINTS:<br>**3 marks**
20	What could be the possible diagnosis?	**0.75 marks** are given for any of these diagnoses:<br>• Noma<br>• Noma stage IV<br>• Noma scarring stage
21	What treatment would you give?	Treatment and advice **can score a maximum of 2.25 marks**. For every of the following items, 0.25 marks where given:<br>• Removal of necrotic tissue or tissue debridement or local cleaning = 0.25<br>• Amoxicillin or ampicillin or penicillin= 0.25<br>• Metronidazole = 0.25<br>• Gentamycin = 0.25<br>• Referral = 0.25<br>• Wound dressing = 0.25<br>• Nutritional support, including references to specific measures e.g. multivitamins = 0.25

22	What advice would you give?	• Nutritional support, including references to specific measures e.g. multivitamins = 0.25 (only if not mentioned in treatment already)<br>• Adhere to treatment = 0.25<br>• Maintain regular appointments to health center or dentist

KNOWLEDGE OF THE DISEASE

	**Knowledge of the disease**	**MAXIMUM SCORE: 12.4 MARKS**
23	Have you ever heard of noma? □ Yes ☐ No<br><br>If **YES**, you've heard of it:	
24	How did you first hear about noma? (Select all that apply)<br>□ During education<br>□ Through the media<br>□ Through your work<br>□ Other:	
25	Which part of the body is affected by the noma? (choose one):<br>□ Foot<br>□ Abdomen<br>X Orofacial region<br>□ I don't know	1 mark
26	Order the phases of the noma with numbers:	1 mark

	4 Gangrene<br>2 Acute necrotising gingivitis<br>6 Sequelae<br>1 Simple gingivitis<br>5 Scarring<br>3 Oedema<br>☐ I don't know	
27	Can noma be prevented? (select one)<br>X Yes ☐ No ☐ Not sure	1 mark
28	What is the mortality rate for noma reported by the WHO? (select one)<br>☐ 10%<br>☐ 50%<br>X 90%<br>☐ I don't know	1 mark
29	How long does it take for a noma to develop from gingivitis to gangrene? (select one)<br>☐ 1 year<br>☐ 3 months<br>X Less than two weeks<br>☐ I'm not sure	1 mark
30	Is malnutrition a reported risk factor for noma? (select one)<br>X Yes ☐ No ☐ Not sure	1 mark
31	Is high blood pressure a risk factor for noma? (select one)<br>☐ Yes X No ☐ Not sure	1 mark
32	Is poor oral hygiene a risk factor for noma? (select one)<br>X Yes ☐ No ☐ Not sure	1 mark
33	Is smoking a reported risk factor for noma? (select one)<br>X Yes ☐ No ☐ Not sure	1 mark
34	I think the noma is caused by: (select one)	1 mark if either chosen

	☐ Virus<br>X Bacteria<br>X Unknown	
35	Is noma contagious? (select one)<br>☐ Yes X No ☐ Not sure	1 mark
36	What should always be done when treating noma?<br>Mouthwash: X Yes ☐ No ☐ I don't know<br>Give as many xima as possible: ☐ Yes X No ☐ I don't know<br>Local disinfection: X Yes ☐ No ☐ Don't know<br>Antibiotic therapy: X Yes ☐ No ☐ Don't know<br>Give additional food with vitamins: X Yes ☐ No ☐ Don't know	0.1 marks for each item correctly answered, maximum of 0.5 marks
37	In your opinion, which of the following is the best way to prevent noma in a patient? (Select all that apply)<br>X Improve oral hygiene<br>X Quitting smoking<br>X Routine childhood vaccination<br>X Stop eating chicken<br>X Improving nutritional status<br>☐ I'm not sure	0.1 marks for each item correctly answered, maximum of 0.5 marks
38	What is the best way to plan for the prevention of noma in society? (Select all that apply)<br>X Improving access to healthcare<br>X Improving referral systems between health centres<br>X Establish partnerships with traditional healers<br>X Improving living conditions (access to drinking water, food, etc.)<br>☐ I don't know	0.1 marks for each item correctly answered, maximum of 0.4 marks

Researcher shows the informative poster

	Knowledge of the disease
39	Have you ever seen such a case?<br>☐ Yes:<br>How many?<br>When each (month and year)? |_|_| - |_|_|_| ☐ No<br><br>*If you have seen a case, tell the researcher so that they can find it and apply the "NOMA IDENTIFIED CASE INFORMATION" questionnaire.*
40	At your health centre you have regular access to:<br>Antibiotics: ☐ Yes ☐ No ☐ Don't know<br>Wound debridement: ☐ Yes ☐ No ☐ Don't know<br>Nutritious foods and vitamins: ☐ Yes ☐ No ☐ I don't know<br>Telephone network: ☐ Yes ☐ No ☐ Don't know
41	Would you be interested in taking part in a training course to learn more about noma?<br>☐ Yes ☐ No ☐ I don't want to answer
42	Which of the following methods would be best for learning more about noma? (Select all that apply)<br>☐ Internet-based interactive courses<br>☐ Guidelines for tablet or smartphone applications<br>☐ Guidelines in written format<br>☐ Weekend courses run by experts<br>☐ More data on local noma studies<br>☐ Be involved in clinical research<br>☐ I'm not sure
