# Supplementary material for: Knowledge, attitudes and practices of healthcare workers towards noma in Zambezia, Mozambique
Source: PLoS Negl Trop Dis. 2025 Mar 19;19(3):e0012939. doi: 10.1371/journal.pntd.0012939 (PMC11952756; doi:10.1371/journal.pntd.0012939)
Supplement: S2 File — (PDF) [file pntd.0012939.s002.pdf]

Conhecimentos, atitudes e práticas dos profissionais de saúde em relação ao Noma na Zambézia, Moçambique

Marta Ribes[1,2]*, Fizaa Halani[2], Abdala Atumane[3], Milagre Andurage[4], Eldo Elobolobo [5,6], Gemma Moncunill[1,2,7], Romina Ríos-Blanco[2,8], Tairo Sumine[9], Luis Transval[9], Fernando Padama[3]†, Carlos Chaccour[1,7,10]†

†Contribuintes iguais

[1] ISGlobal, Barcelona, Espanha

[2] Facultat de Medicina i Ciències de la Salut, Universitat de Barcelona (UB), Barcelona, Espanha

[3] Serviço Provincial de Saúde da Zambézia, Moçambique

[4] Hospital Central de Quelimane, Moçambique

[5] Associancão Silver Lining, Moçambique

[6] Databrew, Moçambique

[7] CIBER de Enfermedades Infecciosas, Madrid, Espanha

[8] Instituto de Medicina Tropical Alexander von Humboldt, Universidad Peruana Cayetano Heredia, San Martin de Porres, Perú

[9] Hospital Geral de Quelimane,

[10] Universidad de Navarra, Pamplona, Espanha

*Autor correspondente

Correio eletrónico e endereço do autor correspondente:marta.ribes@isglobal.org . Carrer Rosselló 171, entresol. 08036 Barcelona, Espanha

Resumo

Antecedentes

O Noma é uma doença tropical negligenciada que afecta sobretudo crianças que vivem na pobreza. Apesar de ser evitável e tratável com medicamentos facilmente acessíveis, estima-se que 90% dos doentes morrem devido à falta de acesso a cuidados rápidos e adequados.

Metodologia e principais conclusões

As unidades sanitárias primárias, secundárias e quaternárias foram visitadas numa base de amostragem de conveniência na província da Zambézia, centro de Moçambique. Os profissionais de saúde foram convidados a participar numa base de amostragem por quotas, e foi-lhes aplicado um questionário que incluía perguntas abertas e fechadas que avaliavam as suas práticas relacionadas com a saúde oral, os seus conhecimentos teóricos sobre o noma e as suas atitudes em relação a receber uma formação em noma.

Um total de 41 profissionais de saúde de 23 unidades de saúde diferentes participaram do estudo. Destes, 59% conheciam o noma, e 26,8% relataram ter atendido pessoalmente um paciente com noma agudo. No entanto, os seus conhecimentos sobre as caraterísticas clínicas ou o tratamento do noma eram fracos, especialmente nas suas fases iniciais. Apenas 12% diagnosticaram corretamente o noma na fase 1 e 5% na fase 2. Os profissionais de nível universitário tinham um conhecimento significativamente melhor da doença do que os enfermeiros e técnicos. Todos os participantes manifestaram interesse em receber formação específica sobre o noma.

Conclusões

As competências de gestão do noma na Zambézia são extremamente baixas, particularmente entre os enfermeiros e técnicos médicos, que servem como o primeiro ponto de cuidados para os pacientes com noma. Há uma necessidade urgente de implementar programas de formação

abrangentes em todos os níveis dos prestadores de cuidados de saúde moçambicanos, para evitar mais mortes evitáveis e reduzir os resultados graves associados ao atraso no tratamento.

Resumo do autor

O noma é uma doença que afecta sobretudo crianças que vivem na pobreza. Começa por ser uma simples infeção das gengivas que, sem tratamento, evolui rapidamente para a morte dos tecidos circundantes, incluindo a pele e os ossos. Os sobreviventes enfrentam frequentemente desfigurações faciais graves, que afectam o seu bem-estar, desde a alimentação e a fala até à socialização. No entanto, o noma pode ser evitado. Se os profissionais de saúde pudessem reconhecê-lo precocemente, um simples tratamento com antibióticos, cuidados com as feridas e uma nutrição adequada - coisas que estão amplamente disponíveis - poderiam parar a doença antes que ela causasse tais danos

Por esta razão, quisemos saber o nível de conhecimento do noma entre os profissionais de saúde na Zambézia, Moçambique. Descobrimos que, embora 59% deles tivessem ouvido falar do noma e 26,8% tivessem tratado um paciente, muito poucos conseguiam diagnosticá-lo ou tratá-lo suficientemente cedo para evitar as suas consequências irreversíveis, ou estavam conscientes da sua letalidade e rápida progressão. Isto é semelhante às conclusões na Zâmbia e no Burkina Faso, onde o noma também tem sido negligenciado.

Este facto indica uma necessidade urgente de e de formação dos profissionais de saúde. O problema não está na falta de acesso ao tratamento, mas sim na falta de acesso a um diagnóstico atempado.

Introdução

O noma (*cancrum oris*) é uma gangrena orofacial de progressão rápida, que afecta sobretudo crianças que vivem na pobreza e, menos frequentemente, adultos imunocomprometidos. Apesar de ser fácil e rapidamente tratável nas suas fases iniciais com antibióticos, apoio nutricional e desbridamento da ferida, a maioria dos casos não é diagnosticada e, por conseguinte, não é tratada, levando a uma mortalidade estimada de 90% em apenas duas semanas[1,2]. A maioria dos sobreviventes da fase aguda vive com desfiguração facial para toda a vida, o que leva a enormes consequências sociais e funcionais, incluindo estigma, isolamento e dificuldades em comer, falar ou ver[1-3].

A etiologia do Noma é ainda desconhecida, embora se pense que resulte de um desequilíbrio da microbiota oral[4,5]. Não é contagioso, nem recorrente, e os factores de risco mais frequentemente relatados são a desnutrição, a má higiene oral e ter sofrido recentemente de outra infeção, como o sarampo ou a malária, nos três meses anteriores.[1] A sua epidemiologia atual não é completamente compreendida, uma vez que existe uma notável falta de relatórios[6]. No entanto, pensa-se que ocorre onde quer que haja pobreza, tal como corroborado por Srour et al. e pela nossa equipa, que encontraram sobreviventes de noma no Laos[7] e em Moçambique, respetivamente, países que não tinham reportado qualquer caso na literatura científica.

A progressão do Noma foi categorizada pela Organização Mundial de Saúde (OMS) em cinco estágios[2], precedidos pelo sinal de alerta ou estágio 0, que corresponde a uma gengivite simples. O estádio 1 descreve uma gengivite necrosante aguda, caracterizada por hálito fétido, ulceração de uma ou mais papilas interdentárias com dor, sangramento espontâneo das gengivas e salivação excessiva. Nesta fase, a administração de uma combinação de amoxicilina oral, metronidazol, acompanhada de analgésicos, tratamento e desbridamento de feridas e apoio nutricional, pára a progressão da doença. Na ausência de tratamento adequado, segue-

se rapidamente a fase 2, caracterizada por um edema no lado exterior de uma úlcera interna que se estende rapidamente, acompanhado de dor intensa e febre alta. O tratamento consiste em antibióticos intravenosos (amoxicilina-ácido clavulânico, penicilina ou ampicilina, com gentamicina e metronidazol), hidratação e nutrição e desbridamento. Até esta fase a doença é reversível; no entanto, na ausência de tratamento, numa questão de horas a dias, evolui para a fase 3 ou fase gangrenosa. Nesta fase, o edema progride para necrose, afectando os tecidos moles e duros, que acabam por se desprender, deixando uma perfuração na zona afetada. Nesta altura, o tratamento do doente consiste no que foi referido anteriormente, com a prioridade de estabilizar o doente. A morte ocorre geralmente nesta fase devido a sépsis, broncoaspiração ou desnutrição. O estádio 4 descreve o processo cicatricial após a paragem da necrose e o estádio 5 refere-se às sequelas crónicas, apenas reversíveis com cirurgia especializada.[8]

O noma só foi oficialmente reconhecido como uma Doença Tropical Negligenciada (DTN) em dezembro de 2023.[9] De facto, a falta de sensibilização tem rodeado o noma desde o meio académico até aos sistemas de saúde.[10] O noma está ausente da maioria dos currículos e das políticas de saúde pública. Como resultado, o conhecimento dos profissionais de saúde está longe de ser ótimo. Até onde sabemos, quatro estudos de pesquisa avaliaram o nível de conhecimento e competência prática dos profissionais de saúde sobre o noma. Em 2009, no distrito de Serenje, na Zâmbia, entre uma coorte de 35 profissionais de saúde com nível pré-universitário, Ahlgren et al. constataram que 54% tinham ouvido falar da doença e todos eles tinham um nível subóptimo ou muito baixo de competência prática global e dois terços, um nível muito baixo de conhecimentos teóricos.[11] Em 2009, no distrito de Nouna, no Burquina Faso, Brattström-Stolt et al. aplicaram um questionário estruturado a 76 enfermeiros e auxiliares de enfermagem e concluíram que 91 % deles tinham ouvido falar do noma, mas 70 % tinham competências práticas subóptimas ou muito baixas, enquanto metade dos participantes tinha conhecimentos teóricos bons ou óptimos. Um subgrupo de enfermeiros

que recebeu uma formação de dois dias tinha um melhor nível de conhecimento do que os seus pares.[12] Em 2019, Bala et al. avaliaram 156 profissionais do Usmanu Danfodiyo University Teaching Hospital, uma instituição terciária no estado de Sokoto, na Nigéria, localizada a apenas cinco quilómetros do Noma Children Hospital,[13] e encontraram uma taxa de sensibilização de 79%.[14] Em 2022, a mesma equipa entrevistou 251 profissionais de saúde primários e verificou que 83,7% tinham conhecimento do noma, e entre eles, 81,4% tinham visto ou gerido um caso de noma, e 43,4% tinham encaminhado um caso para o Noma Children Hospital.[(15]

Devido à natureza de progressão rápida da doença, é necessário um diagnóstico correto desde o ponto de entrada dos cuidados. Na experiência deste grupo, e conforme relatado por Farley et al.[16] e Baratti-Mayer et al.,[17] aproximadamente 30 a 40% dos pacientes com noma consultam primeiro um curandeiro tradicional. Estes mesmos estudos descobriram que apenas 10,5% dos curandeiros tradicionais no Mali tinham algum conhecimento sobre o noma,[17] enquanto no estado de Sokoto, na Nigéria, um estudo qualitativo que utilizou entrevistas aprofundadas descobriu que os curandeiros tradicionais reconheciam que atendiam doentes com noma, especialmente nas suas fases iniciais, mas não sabiam de que doença se tratava.[16]

Em Moçambique, o Sistema de Informação de Saúde para Monitoria e Avaliação não inclui atualmente o noma como uma doença, nem há casos relatados na literatura científica[6]. No entanto, num período de cinco semanas, a nossa equipa conseguiu identificar 21 sobreviventes de noma e dois casos agudos na Província da Zambézia, evidenciando a sua endemicidade

Os enfermeiros e os técnicos de medicina constituem a maioria da mão de obra no sector da saúde em Moçambique. A sua formação tem a duração de dois anos e dois anos e meio,[18] respetivamente, nos *institutos técnicos médios profissionais* (instituições de ensino pré-universitário). Os seus curricula não incluem o noma, nem este está incluído no curso de

medicina das universidades. Apenas os estomatologistas e os cirurgiões maxilofaciais têm formação sobre doença durante o internato, enquanto os pediatras não têm. No entanto, estes especialistas constituem uma força de trabalho escassa, com rácios nacionais de um estomatologista por cada 88.533 habitantes, e totais nacionais de 14 cirurgiões maxilofaciais e quatro cirurgiões plásticos. Como resultado, apenas 15% dos centros de cuidados de saúde primários (*Unidades Sanitárias*) prestam cuidados de estomatologia, 11% na Zambézia.[19] Finalmente, os curandeiros tradicionais que prestam cuidados tradicionais para doenças, ultrapassam largamente os profissionais médicos, uma vez que por cada médico, existem 50 curandeiros tradicionais.[(20]

O principal objetivo deste estudo foi avaliar o grau de conhecimento sobre o noma entre os profissionais de saúde que trabalham nas unidades sanitárias da Província da Zambézia, centro de Moçambique, centrando-se tanto na compreensão teórica como nas competências práticas de gestão.

Métodos

Local de estudo

O local do estudo foi escolhido com base em relatórios informais anteriores de doentes com noma que viviam na província. A província da Zambézia tem uma população de 5,1 milhões de habitantes distribuídos por 22 distritos[21]. O nível mais elevado de cuidados de saúde é prestado pelo Hospital Central de Quelimane, situado na capital. Este é o único centro de quarto nível na província, que alberga o único cirurgião maxilofacial da província. Não existe nenhum hospital de nível terciário na Zambézia. O nível secundário de cuidados é prestado pelo Hospital Geral de Quelimane, que também presta alguns cuidados especializados, incluindo cirurgia básica, e pelos hospitais distritais ou rurais, situados na cidade principal de cada distrito. O nível primário é composto por centros de saúde e postos de saúde, servidos principalmente por enfermeiros e técnicos, bem como por um total de 470 agentes

comunitários de saúde (*Agentes Polivalentes de Saúde*) que prestam cuidados ao domicílio na província. Existem 6,2 enfermeiros, médicos, parteiras, estomatologistas ou farmacêuticos por 10.000 habitantes na Zambézia,[22] o que está muito abaixo do limiar mínimo de densidade da OMS de 22,8 profissionais de saúde por 10.000 habitantes.[23] Em 2016, 18.9% da força de trabalho médica eram enfermeiros de saúde materno-infantil, 31% enfermeiros generalistas, 14,8% técnicos médicos, 10% técnicos de saúde pública, 7,4% laboratoristas, 6,8% farmacêuticos, 3,1% médicos, 1,5% estomatologistas e 6,4% outros técnicos.[18,22]

Procedimentos

Realizámos um estudo transversal entre janeiro e fevereiro de 2024. Este foi um dos objectivos do projeto "Ecos do Noma", realizado em colaboração com o Serviço Provincial de Saúde da Zambézia , visando fornecer a primeira evidência científica da presença do noma em Moçambique. Aproveitando as visitas a 13 distritos da Zambézia à procura de sobreviventes de noma, visitámos unidades sanitárias numa base de amostragem de conveniência. Um a três provedores de saúde por unidade sanitária foram convidados a participar no estudo, escolhidos numa base de amostragem por quotas, com o objetivo de obter uma amostra diversificada em termos profissionais.

Após a assinatura do consentimento informado, um questionário CAP, incluindo perguntas fechadas e abertas, foi lido em voz alta, em português, individualmente para cada participante e preenchido por um investigador treinado. Finalmente, os profissionais de saúde foram informados sobre o diagnóstico, o tratamento e a prevenção do noma através de um cartaz que foi deixado à sua disposição, juntamente com um número de telefone para encorajar a futura notificação de casos suspeitos.

O questionário CAP (ver Material Suplementar) foi desenvolvido com base nos anteriormente utilizados na Zâmbia[11], Burkina Faso[12], e pelos *Médicos Sem Fronteiras* no Estado de Sokoto, Nigéria. Continha perguntas fechadas sobre dados demográficos, práticas relacionadas com a

saúde oral, conhecimentos teóricos sobre o noma e atitudes em relação a receber formação sobre o noma. A competência prática ou de gestão foi avaliada com perguntas abertas relativas ao diagnóstico, tratamento e recomendações para três casos práticos de noma, que eram iguais aos casos simulados A, B e D no questionário de Ahlgren et al.[11]

Análise estatística

Os dados foram digitalizados independentemente por dois codificadores usando a ferramenta Double Data Entry do REDCap[24,25] e comparados quanto a incongruências por uma terceira pessoa. As tabelas e os cálculos foram efectuados com o software R versão 4.3.3. As variáveis categóricas são apresentadas como frequências e percentagens, e as variáveis contínuas paramétricas como médias com desvios-padrão (DP) e as variáveis contínuas não paramétricas como medianas com intervalo interquartil (IQR)

Os casos práticos foram classificados em 3 pontos e as perguntas sobre conhecimentos teóricos sobre o noma foram classificadas num total de 12,4 pontos, conforme indicado no esquema de classificação fornecido em Material suplementar. As notas foram ainda classificadas em "óptima" (>=75% da nota total), "boa" (50 a 74%), "subóptima" (25 a 49%) e "muito baixa" (<25%).

Para avaliar as diferenças entre os níveis de escolaridade, a categoria do ensino secundário foi retirada por ter sido representada por um único participante. Devido às baixas frequências, realizámos o teste exato de Fisher para testar as diferenças nas variáveis categóricas entre os níveis de ensino. Para as variáveis numéricas, efectuámos primeiro o teste de Shapiro-Wilk para verificar a normalidade. Quando os dados não se distribuíam normalmente, aplicámos o teste U de Mann-Whitney. Para o único caso em que os dados tinham uma distribuição normal, verificámos a sua variância e, uma vez que era igual, calculámos um teste T.

Todos os testes efectuados são comunicados.

Aprovação ética

O estudo foi revisto e aprovado pelo Comité Institucional de Bioética para Saúde da Zambézia sob o número 219/CIBS-Z/23. O Serviço Provincial de Saúde da Zambézia validou o estudo e garantiu o acesso às unidades sanitárias da província. Todos os participantes deram o seu consentimento informado.

Resultados

Caraterísticas dos participantes no estudo

Um total de 41 profissionais de saúde participaram no estudo, trabalhando em 23 diferentes unidades sanitárias de 12 distritos diferentes na Zambézia (Alto Molocué, Gurué, Ile, Maganda da Costa, Mocuba, Mocubela, Mopeia, Morrumbala, Namacurra, Nicoadala, Pebane e Quelimane) (Figura 1). Todos os profissionais convidados a participar aceitaram, exceto um. Quarenta e um por cento (17/41) trabalhavam em hospitais de nível quaternário ou secundário e 59% (24/41) em centros de cuidados primários. Um tinha o nível secundário, 71% (29/41) o nível pré-universitário e 27% o nível universitário, entre os quais sete eram estomatologistas, três eram médicos e um era nutricionista (Tabela 1)

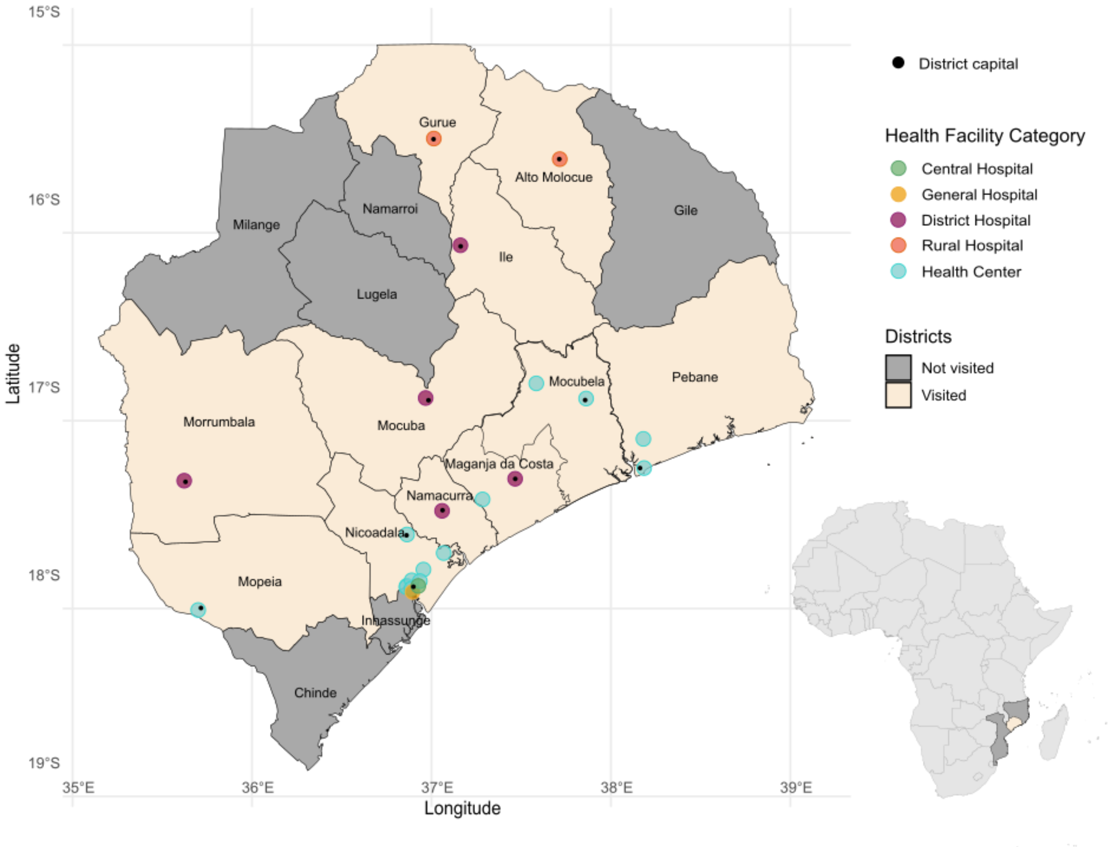

Figura 1. **Mapa do local de estudo.** Os distritos visitados estão representados em marfim e as suas capitais estão assinaladas com pontos pretos. Os pontos coloridos indicam as diferentes geolocalizações e categorias de unidades sanitárias visitadas.

De acordo com os seus auto-relatos, 32% dos participantes atendiam menos de cinco crianças por dia, 28% de cinco a dez, 2% de 11 a 15, 10% de 16 a 24 e 20% mais de 24. Estas proporções não foram estatisticamente significativas entre os níveis de ensino, embora os profissionais de nível universitário atendessem menos pacientes por dia do que os de nível pré-universitário. A maioria dos participantes trabalhava em unidades de saúde onde menos de 50 crianças com menos de cinco anos procuravam cuidados orais num mês. A mediana do número de crianças atendidas com feridas orais por profissional de saúde e por ano foi de 10 (IQR = 23), com edema da bochecha foi de 5,5 (IQR = 17,75) e com necrose dos tecidos faciais foi de 0 (IQR =

1). Os profissionais de nível pré-universitário viram significativamente mais crianças com feridas orais anualmente do que os profissionais de nível pré-universitário ($p<.05$).

**Variável**	**N***	**% ou mediana (IQR)**
**Género**	**41**	
Feminino	21	51%
Masculino	20	49%
**Tipo de estabelecimento de saúde**	**41**	
Unidades Sanitárias	24	59%
Hospital Geral (nível secundário)	3	7%
Hospital Distrital (nível secundário)	11	27%
Hospital rural (nível secundário)	2	5%
Hospital Central (nível quaternário)	1	2%
**Tipo de profissional de saúde**	**41**	
**Nível secundário**	**1**	**2%**
Enfermeiro assistente (*Agente de medicina*)	1	2%
**Nível pré-universitário**	**29**	**71%**
Técnico de nutrição	1	2%
Técnico de estomatologia	5	12%
Técnico de medicina	11	27%
Enfermeira	5	12%
Enfermeiras de saúde materno-infantil (parteiras)	6	15%
Estudante de enfermagem de estomatologia	1	2%
**Nível universitário**	**11**	**27%**
Nutricionista	1	2%
Médico	3	7%
Estomatologista (dentista)	7	17%
**Número de crianças com menos de 5 anos assistidas por dia**	**40**	
< 5	13	32%
5 - 10	11	28%
11 - 15	1	2%
16 - 24	4	10%
> 24	8	20%
Não sei	3	8%
**Número de crianças com menos de 5 anos que procuram cuidados orais na sua unidade de saúde por mês**	**38**	
<= 25	22	58%
26 - 50	5	13%
51 - 75	3	8%
76 - 99	2	5%
>= 100	1	3%
Não sei	5	13%
**Número de crianças atendidas pelo profissional por ano com sangramento, inchaço ou ulceração gengival**	**32**	10 (23)
**Número de crianças atendidas pelo profissional por ano com edema facial**	**34**	5.5 (17.75)

**Número de crianças atendidas pelo profissional por ano com necrose de tecidos faciais**	**30**	0 (1)
**Alguma vez assistiu ou viu um doente com noma**	**41**	
Sim	23	56%
Não	18	41%

Tabela 1. Caraterísticas dos prestadores de cuidados de saúde que participaram no estudo.

*Total de participantes que podiam fornecer as informações e eram aplicáveis para responder

Práticas

Os participantes foram questionados sobre as suas práticas de exame oral quando atendiam determinadas condições. Entre os que atendiam crianças, quando se deparavam com uma criança com malária, 61% (22/36) disseram que examinavam a boca, 31% (11/36) disseram que não o faziam e três não tinham a certeza. Ao atender uma criança malnutrida, 80% (28/35) declararam verificar a boca, contra 20% (7/35) que não o fizeram. Por último, ao atender uma criança com VIH, 89% (31/35) disseram que verificariam a boca, contra 9% (3/35) que disseram que não o fariam e um que não tinha a certeza. Estas proporções não diferem significativamente entre o nível pré-universitário e o universitário, embora sejam consistentemente mais elevadas no último grupo.

Conhecimento

Competências de gestão

Todos os participantes foram confrontados com três casos práticos antes de ser feita qualquer menção ao noma, e foram-lhes colocadas perguntas abertas sobre o seu diagnóstico, tratamento e aconselhamento (ver questionário e esquema de pontuação nos Materiais Suplementares). O primeiro caso foi descrito como "Uma mãe procura cuidados para o seu filho de dois anos que tem hemorragia gengival e mau hálito" e mostrava uma imagem dos incisivos primários da criança com hemorragia visível e inflamação da gengiva vestibular, perda

de papilas interdentárias e algum tecido necrótico cinzento, exibindo sinais de gengivite ulcerativa necrosante aguda (ANUG ou noma fase 1). Doze por cento diagnosticaram corretamente o caso como gengivite necrosante ou gengivite necrosante aguda ou gengivite ulcerativa. Os diagnósticos incorrectos incluíram gengivite simples (mencionada por 22 participantes em 41), cáries (5/41), infeção por candidíase (4/41), aftas (2/41), amigdalite (2/41), e outros mencionaram varíola, sarcoma de Kaposi, herpes, abcesso, VIH ou malária; e quatro não sabiam. Quando questionados sobre o tratamento que dariam, 61% disseram que dariam amoxicilina e 68% dariam analgésicos; enquanto apenas 7% mencionaram metronidazol, 15% desbridamento de feridas e 20% apoio nutricional. Sete mencionaram que administrariam penicilina e sete mencionaram nistatina, um antifúngico. A higiene oral foi o conselho mais recomendado, mencionado por 90% dos participantes, seguido de bochechos com água salgada (46% recomendaram-nos), evitar alimentos quentes (12%) e aderir ao tratamento (5%). A mediana da nota de tratamento e aconselhamento para o caso 1 foi de 0,75 em 2,25 (IQR=0,75) e a mediana da nota total quando se adicionam os pontos de diagnóstico foi de 0,75 em 3 (IQR=0,75). Os prestadores de cuidados de saúde com nível universitário foram, em geral, mais corretos nas suas respostas, 27% dos quais diagnosticaram corretamente a doença em comparação com 7% dos prestadores com nível pré-universitário. Oitenta e dois por cento prescreveram bochechos com água salgada, contra apenas 34% dos que tinham um diploma pré-universitário ($p<.05$). Da mesma forma, a mediana da nota de tratamento e aconselhamento e a nota total foram 0,25 pontos mais altas, embora a diferença não tenha atingido significância estatística (ver Tabela 2).

O caso 2 foi descrito como "Um doente de 4 anos de idade com febre e lábios e bochecha inflamados" e retratava uma criança com um edema considerável na bochecha esquerda, atingindo a comissura e a região suborbital (noma estádio 2). Apenas dois participantes (5%) diagnosticaram corretamente o caso como noma. Trinta e dois por cento diagnosticaram incorretamente um abcesso, 22% celulite e 20% papeira. Outros diagnósticos mencionados

foram anemia, conjuntivite, desnutrição, gengivite, miosite, mastoidite, cárie ou malária. À semelhança do caso 1, 59% dos participantes recomendariam a administração de amoxicilina, ampicilina ou penicilina quando se apresentasse uma criança como a descrita no caso 2, e 68% prescreveriam analgésicos. Dezassete por cento administrariam metronidazol, 12% gentamicina, 20% apoio nutricional e 7% reidratação. É importante referir que apenas 24% encaminhariam para outro centro e 12% aconselhariam a família a aderir ao tratamento. A mediana da pontuação relativa ao tratamento e ao aconselhamento foi de 0,5 em 2,25 (IQR=0,25) e a mediana da pontuação total, somando os pontos de diagnóstico, foi de 0,5 em 3 (IQR=0,25). Os profissionais com formação universitária tiveram um melhor desempenho, embora as diferenças não tenham sido estatisticamente significativas, exceto no que diz respeito à recomendação de dar apoio nutricional, que foi dada por 45% dos profissionais com formação universitária, contra 10% dos profissionais com nível pré-universitário. Tal como no caso 1, a mediana da nota de tratamento e aconselhamento e a nota total foram 0,25 pontos superiores para os profissionais com formação universitária, embora a diferença não tenha atingido significado estatístico.

A imagem do Caso 3 era acompanhada pela descrição "Paciente de 25 anos com tecido gangrenado e um buraco na bochecha" e mostrava uma mulher jovem com perda significativa de tecido mole, estendendo-se vários centímetros da comissura esquerda em direção ao ângulo da mandíbula. Isto incluía a bochecha inferior, bem como o tecido labial superior e inferior distal dos pré-molares. O defeito expôs a dentição subjacente e a cavidade oral, na qual faltam todos os molares inferiores e se observa recessão gengival à volta dos dentes remanescentes. Podem ser observadas cicatrizes elevadas significativas à volta do defeito, que são mais proeminentes à volta da mandíbula (noma de estádio 4). Quarenta e seis por cento diagnosticaram corretamente o noma, 79% entre os que já tinham ouvido falar da doença anteriormente. Entre os restantes, três diagnosticaram uma úlcera, dois uma gangrena oral, dois um abcesso, um um cancro oral, um uma infeção e 13 não sabiam. Sessenta e três por

cento recomendaram o encaminhamento do doente para um especialista, 22% recomendaram a administração de amoxicilina, ampicilina ou penicilina, 15% de metronidazol, 15% de gentamicina e 15% a remoção do tecido necrótico. Relativamente aos conselhos ao doente ou à sua família, 37% recomendaram a ida regular à unidade de saúde para fazer o penso da ferida, 12% aconselharam a nutrição, 10% a adesão ao tratamento e 5% a visita regular às unidades de saúde. A mediana das classificações de tratamento e aconselhamento foi de 0,5 em 2,25 (IQR=0,5) e a mediana da classificação total, somando os pontos de diagnóstico, foi de 1 em 3 (IQR= 1). Oitenta e dois por cento dos profissionais com um diploma universitário diagnosticaram corretamente o noma, enquanto apenas 34% dos
os de nível pré-universitário fizeram-no (p<.05). Do mesmo modo, os profissionais de nível universitário recomendaram, numa proporção significativamente mais elevada, o encaminhamento do doente (100% vs 52%, p<.05), e aconselharam sobre nutrição (36% vs 3%, p<.05). Pelo contrário, os profissionais de nível pré-universitário tiveram um desempenho ligeiramente superior em algumas recomendações de tratamento, como a administração de gentamicina, ou no aconselhamento de visitas regulares às unidades de saúde, e recomendaram significativamente mais a realização de pensos (9% vs 48%, p<.05). A nota total para o terceiro caso foi significativamente mais elevada nos profissionais de nível universitário, que obtiveram 1,25 em 3 (IQR=0,65) contra 0,75 em 3 (IQR=0,75) nos profissionais de nível pré-universitário (Tabela 2)

Ao somar as pontuações dos três casos práticos, a pontuação média foi de 2,25 em 9 (DP=2,1). Em geral, os profissionais de nível universitário tinham uma competência de gestão significativamente melhor do que os de nível pré-universitário, com uma pontuação média de 3,25 em 9, contra 2 em 9 (p<.05) (Tabela 2). A melhor competência de gestão foi encontrada entre os técnicos de estomatologia (nota média de 3,5), seguidos pelos estomatologistas (média de 3,4), médicos (média de 3,2), técnicos de medicina (média de 2,2), técnicos de

nutrição (média de 2), enfermeiros (média de 1,7), parteiras (média de 1,3) e, por último, nutricionistas e agentes comunitários de saúde (média de 1).

Ao classificar as suas notas em quartis, entre os profissionais pré-universitários, a grande maioria (72%) tinha uma competência de gestão "muito baixa", 24% "subóptima" e 3% "boa"; enquanto a maioria dos profissionais de nível universitário tinha uma competência de gestão "subóptima" (64%), 18% "boa" e 18% "muito baixa". Nenhum tinha uma competência de gestão "óptima".

	Total			Nível pré-universitário			Nível universitário				
**Variável**	N	Mediana/%	IQR	N	Mediana/%	IQR	N	Mediana/%	IQR	Teste estatístico	Valor de p
**CASO 1**	41			29			11				
**Diagnóstico correto** (ANUG / Noma fase 1 / Gengivite ulcerosa / Gengivite necrosante)	5	12%		2	7%		3	27%		4.8	0.12
**Tratamento e aconselhamento recomendados**											
Amoxicilina	25	61%		18	62%		7	64%		1.1	1.00
Metronidazol	3	7%		2	7%		1	9%		1.3	1.00
Desbridamento de feridas	6	15%		4	14%		2	18%		1.4	1.00
Apoio nutricional	8	20%		5	17%		3	27%		1.8	0.66
Analgésicos	28	68%		19	66%		8	73%		1.4	1.00
Higiene oral	37	90%		25	86%		11	100%		Inf	0.56
Lavagens com água salgada	19	46%		10	34%		9	82%		**8.1**	**0.01**
Adesão ao tratamento	2	5%		2	7%		0	0%		0.0	1.00
Para evitar alimentos quentes	5	12%		3	10%		2	18%		1.9	0.60
Nota de tratamento e aconselhamento (em 2,25)	41	0.75	0.75	29	0.75	0.75	11	1	0.5	108.0	0.11
**Nota total (em 3)**	41	0.75	0.75	29	0.75	0.75	11	1	0.875	97.0	0.06
**CASO 2**	41			29			11				
**Diagnóstico correto** (noma / estádio 3 / estádio de edema do noma)	2	5%		1	3%		1	9%		2.7	0.48
**Tratamento e aconselhamento recomendados**											
Amoxicilina/Ampicilina/Penicilina	24	59%		16	55%		7	64%		1.4	0.73
Metronidazol	7	17%		3	10%		4	36%		4.7	0.08
Gentamicina	5	12%		3	10%		2	18%		1.9	0.60
Apoio nutricional	8	20%		3	10%		5	45%		**6.8**	**0.02**
Re-hidratação	3	7%		3	10%		0	0%		0.0	0.55
Analgésicos	28	68%		21	72%		6	55%		0.5	0.45
Referência	10	24%		7	24%		3	27%		1.2	1.00
Adesão ao tratamento	5	12%		5	17%		0	0%		0.0	0.30
Nota de tratamento e aconselhamento (em 2,25)	41	0.5	0.25	29	0.5	0.25	11	0.75	0.5	139.0	0.53
**Nota total (em 3)**	41	0.5	0.25	9	0.5	0.25	11	0.75	0.5	139.5	0.54
**CASO 3**	41			29			11				
**Diagnóstico** (noma / estádio 4 / estádio de cicatrização do noma)	19	46%		10	34%		9	82%		**8.1**	**0.01**
**Tratamento e aconselhamento recomendados**											
Remoção de tecido necrótico ou desbridamento da ferida	6	15%		3	10%		3	27%		31.4	0.32
Referência	26	63%		15	52%		11	100%		**inf**	**0.00**
Amoxicilina/Ampicilina/Penicilina	9	22%		6	21%		3	27%		1.4	0.69
Metronidazol	6	15%		4	14%		2	18%		1.4	1.00
Gentamicina	6	15%		5	17%		1	9%		0.5	1.00

Adesão ao tratamento	4	10%		3	10%		1	9%		0.9	1.00
Pensos para feridas	15	37%		14	48%		1	9%		**0.1**	**0.03**
Apoio nutricional	5	12%		1	3%		4	36%		**14.6**	**0.02**
Visitas regulares ao estabelecimento de saúde	2	5%		2	7%		0	0%		0.0	1.00
Nota de tratamento e aconselhamento (em 2,25)	41	0.5	0.5	29	0.25	0.5	11	0.5	0.625	125.0	0.28
**Nota total (em 3)**	41	1	1	29	0.75	0.75	11	1.25	0.65	**80.0**	**0.02**
**Nota total de competências de gestão (em 9)**	**41**	**2.25**	**1.5**	**29**	**2**	**1**	**11**	**3.25**	**1.375**	**85.5**	**0.03**

Tabela 2. Desempenho dos participantes no diagnóstico e prescrição de tratamento e aconselhamento quando confrontados com três casos práticos de doentes com noma. A um diagnóstico correto foi atribuído 0,75 pontos e a cada tratamento ou conselho mencionado foi atribuído 0,25 pontos (exceto o encaminhamento, que obteve 0,5 pontos). Os dados são apresentados como totais e segregados por nível de escolaridade mais elevado. O nível secundário foi omitido por ter sido representado apenas por um participante. Para avaliar as diferenças entre os dois níveis de ensino, foi utilizado o teste exato de Fisher para as variáveis categóricas e o teste U de Mann-Whitney para as variáveis numéricas, exceto para as variáveis com um asterisco (*) que foram testadas com um teste T por serem paramétricas. As estatísticas a negrito são estatisticamente significativas ($p<.05$).

Conhecimentos teóricos sobre o noma

Após os casos práticos, foi perguntado aos participantes se já tinham ouvido falar sobre o noma. Cinqüenta e nove por cento (24/41) conheciam o noma, sendo 52% entre os profissionais de nível pré-universitário (principalmente enfermeiros, técnicos de enfermagem e técnicos em estomatologia) e 82% entre os de nível universitário (Tabela 3). Setenta e um por cento ouviram falar durante os estudos (como técnico médico, técnico de estomatologia, médico, enfermeiro e estomatologista), 12,5% nos meios de comunicação, 21% no trabalho e um durante um curso sobre HIV

Outras perguntas fechadas sobre o conhecimento teórico do noma foram feitas aos 24 participantes que responderam afirmativamente. Todos eles sabiam que o noma afecta a

região orofacial, 83% sabiam que podia ser prevenido, 75% que não era contagioso e 67% que era uma infeção bacteriana. No entanto, apenas 14% sabiam ordenar corretamente os estadios do noma (o erro mais comum foi ordenar o edema antes do ANUG), 17% conheciam a sua taxa de mortalidade e 12% sabiam que evolui em menos de duas semanas (Tabela 3). Quanto aos factores de risco, todos reconheceram a má higiene oral como fator de risco, 96% a desnutrição, 83% o tabagismo e 67% descartaram corretamente a hipertensão arterial como fator de risco. Da mesma forma, o desempenho foi relativamente alto também na identificação de ações preventivas para a doença, com 100% reconhecendo a melhoria da higiene bucal, 96% a melhoria do estado nutricional e 62% reconhecendo a vacinação infantil como medida preventiva. A maioria dos participantes reconheceu corretamente os elixires bucais (78%), a desinfeção da ferida (96%), os antibióticos (96%) e a suplementação vitamínica (96%) como tratamento para o noma. Finalmente, 96% reconheceram a melhoria do acesso aos cuidados de saúde como uma boa medida de saúde pública para prevenir o noma, 88% para melhorar as condições de vida, 75% para melhorar os sistemas de referência entre os centros de saúde e 58% para estabelecer parcerias com curandeiros tradicionais.

Quando comparados os níveis de escolaridade, os que tinham um diploma universitário tiveram um desempenho ligeiramente melhor na maioria das questões, e ultrapassaram significativamente os seus colegas pré-universitários no conhecimento da progressão temporal do noma, uma vez que 33% responderam corretamente que evolui em menos de duas semanas, enquanto nenhum dos pré-universitários conhecia a sua natureza de progressão rápida ($p<.05$). A mediana do conhecimento sobre o noma dos 24 participantes que já tinham ouvido falar sobre a doença foi de 8,75 em 12,4 (DP= 2), 9,1 nos profissionais de nível universitário e 8,2 nos profissionais de nível pré-universitário ($p<.05$). Ao classificar as notas em quartis, entre os profissionais de nível pré-universitário, a grande maioria (73%) tinha um conhecimento teórico "bom", 20% um "ótimo" e 7% "subóptimo". Entre os profissionais de

nível universitário, 56% tinham um conhecimento teórico "bom" e 44% um conhecimento "ótimo".

Variável	Total			Nível pré-universitário			Nível universitário			Teste estatístico	Valor de p
	N	Média/%	SD	N	Mediana/%	SD	N	Média/%	SD		
**Já ouviu falar de noma?**	**41**			**29**			**11**			4.06	0.15
Sim	24	59%		15	52%		9	82%			
Não	17	41%		14	48%		2	18%			
**Que região é afetada pelo noma?**	**24**			**15**			**9**				
Região orofacial	24	100%		15	100%		9	100%			
**É possível ordenar os estádios do noma de acordo com a OMS?**	**24**			**15**			**9**			3.76	0.53
Encomenda correta	3	12.5%		1	7%		2	22%			
Encomenda incorrecta	21	87.5%		14	93%		7	78%			
**O noma pode ser prevenido?**	**24**			**15**			**9**			1.95	1.00
Sim	20	83%		12	80%		8	89%			
Não sei	4	17%		3	20%		1	11%			
**Qual é a mortalidade do noma?**	**24**			**15**			**9**			1.81	0.61
10%*	7	29%		5	33%		2	22%			
90%	4	17%		2	13%		2	22%			
Não sei	13	54%		8	53%		5	56%			
**Quanto tempo é que o noma demora a evoluir de gengivite para gangrena?**	**24**			**15**			**9**			**Inf**	**0.04**
Menos de 2 semanas	3	12%		0	0%		3	33%			
3 meses*	7	29%		4	27%		3	33%			
Um ano*	5	21%		5	33%		0	0%			
Não sei	9	38%		6	40%		3	33%			
**A malnutrição é um fator de risco para o noma?**	**24**			**15**			**9**			Inf	1.00
Sim	23	96%		14	93%		9	100%			
Não sei	1	4%		1	7%		0	0%			
**A tensão arterial elevada é um fator de risco para o noma?**	**24**			**15**			**9**			2.25	0.66
Sim*	3	12%		2	13%		1	11%			
Não	16	67%		9	60%		7	78%			
Não sei	5	21%		4	27%		1	11%			
**A má higiene oral é um fator de risco para o noma?**	**24**			**15**			**9**				
Sim	24	100%		15	100%		9	100%			
**O tabagismo é um fator de risco para o noma?**	**24**			**15**			**9**			0.55	0.61
Sim	20	83%		13	87%		7	78%			
Não*	2	8%		1	7%		1	11%			
Não sei	2	8%		1	7%		1	11%			

**Qual é a etiologia do noma?**	**24**			**15**			**9**			inf	0.51
Bactérias*	16	67%		10	67%		6	67%			
Desconhecido*	6	25%		3	20%		3	33%			
Vírus	2	8%		2	13%		0	0%			
**O noma é contagioso?**	**24**			**15**			**9**			3.80	0.35
Sim*	2	8%		1	7%		1	11%			
Não	18	75%		10	67%		8	89%			
Não sei	4	17%		4	27%		0	0%			
**Nota total dos conhecimentos teóricos** (de 12,4)	**24**	**8.75**	**2**	**15**	**8.2**	**1.85**	**9**	**9.1**	**0.7**	**-2.153#**	**0.04**
**Nota total de conhecimentos** (nota de competências de gestão+ nota de conhecimentos teóricos) (de 21,4)	**24**	**12**	**2.1**	**15**	**11**	**1.8**	**9**	**13**	**1.8**	**-2.705#**	**0.01**

Tabela 3. Desempenho dos participantes quanto ao conhecimento teórico sobre a noma. Os dados são apresentados como totais e segregados por nível de escolaridade mais elevado. O nível secundário foi omitido por ter sido representado por apenas um participante. Para avaliar as diferenças entre os dois níveis de escolaridade, foi utilizado o teste exato de Fisher para as variáveis categóricas e o teste T para as variáveis numéricas, uma vez que eram paramétricas (#). As estatísticas a negrito são estatisticamente significativas (p<.05). As classificações para cada questão estão indicadas no esquema de classificação em anexo. Os * indicam respostas que foram consideradas como uma única categoria (correta ou incorrecta) para o teste estatístico de avaliação das diferenças de desempenho.

Ao somar as pontuações dos casos práticos e do conhecimento teórico sobre o noma, entre aqueles que já tinham ouvido falar sobre o noma antes e, portanto, eram aplicáveis para responder às questões de conhecimento teórico, a nota média foi 12 de 21,4 (DP=2,1). Os profissionais de nível universitário tiveram um desempenho significativamente melhor do que os de nível pré-universitário, com uma nota média de 13 em 21,4, contra 11 em 21,4 (p<0,05) (Tabela 3). Os médicos obtiveram a pontuação mais elevada, com uma média de 14, seguidos pelos estomatologistas (média 13), técnicos de estomatologia (média 12), enfermeiros (média 10) e técnicos de saúde (média 9,9). Os restantes participantes das categorias profissionais nunca tinham ouvido falar do noma. Em relação aos quartis, entre os profissionais de nível pré-universitário, 60% tiveram uma pontuação total "subótima" e 40% "boa"; enquanto a maioria

dos profissionais de nível universitário teve uma pontuação "boa" (78%), um teve uma pontuação "subótima" e um uma pontuação "ótima".

Depois de responderem a todas as perguntas, os profissionais foram informados sobre os sinais e sintomas do noma e o tratamento recomendado. De todos os participantes, 56% (23/41) já tinham visto um caso de noma antes. A pergunta foi feita de forma suficientemente abrangente para incluir ter encontrado um sobrevivente de noma na rua ou como paciente. Onze (26,8%) tinham atendido os próprios doentes durante a fase aguda. Entre eles, quatro eram médicos, duas parteiras, dois técnicos de estomatologia, um enfermeiro, um estomatologista e um técnico de saúde. Nos distritos de Gilé e Alto Molocué, a norte, dois médicos referiram ter atendido crianças de três anos com noma agudo em novembro e abril de 2022, respetivamente. Um técnico de estomatologia do Hospital de Mocuba recordou ter atendido pelo menos 12 casos, sendo o último em 2014, enquanto em 2008 e 2009 era frequente, sendo admitido um novo caso a cada três meses. No Hospital Distrital de Morrumbala um médico recordou ter atendido três casos agudos em simultâneo no Hospital de Nampula por volta de 2017.

Todos os participantes declararam ter antibióticos à sua disposição, embora a escassez tenha sido mencionada por vários deles. Oitenta e dois por cento tinham acesso a desbridamento de feridas e 77% a suplementos alimentares. Todos os participantes que referiram falta de equipamento de desbridamento de feridas provinham de centros de saúde primários e, dos sete participantes que referiram falta de vitaminas, seis provinham de centros de saúde primários e um participante de um hospital rural.

Todos os participantes estavam interessados em receber formação adicional sobre o noma, sendo os métodos preferidos os cursos de fim de semana ministrados por especialistas (63%) e o envolvimento em projectos de investigação (59%). Os menos preferidos foram ter dados sobre estudos locais sobre o noma (32%) e cursos online (39%).

Discussão

Avaliámos os conhecimentos, atitudes e práticas dos profissionais de saúde dos níveis quaternário, secundário e primário na província da Zambézia, no centro de Moçambique. Embora mais de metade deles já tivesse ouvido falar da doença, poucos conheciam a sua elevada taxa de mortalidade ou a sua rápida progressão, ou podiam diagnosticar ou tratar adequadamente nas suas fases iniciais. Os profissionais com grau universitário tinham, em geral, um melhor conhecimento das especificidades do noma e das competências de gestão do que os profissionais com nível pré-universitário, que constituem a maior parte da força de trabalho dos cuidados de saúde na região.

O rastreio oral de rotina para crianças em unidades de saúde foi sugerido como uma estratégia de prevenção chave para o noma.[1] No nosso estudo, a frequência relatada de rastreio oral em pacientes com condições que predispõem ao noma foi relativamente alta, com mais de 80% de taxa de rastreio para pacientes com HIV ou desnutrição, e 61% com malária. Estes valores são comparáveis aos resultados obtidos por Brattström no Burkina Faso[12] e por Ahlgren na Zâmbia.[11] No entanto, o número médio auto-relatado de crianças atendidas com gengivite foi de 10 por ano por participante. Dada a prevalência estimada de 7% de sangramento gengival entre crianças com menos de 12 anos a nível nacional,[26] se os rastreios fossem efetivamente tão elevados, seria de esperar um maior número de casos de gengivite. Por exemplo, um profissional que atendesse 10 crianças por dia, atenderia cerca de 2.300 crianças por ano; assumindo uma taxa de rastreio oral de 60% e uma prevalência de gengivite de 7%, 97 casos de gengivite teriam sido detectados por esse profissional, enquanto a mediana relatada foi de 10. Esta discrepância sugere um possível viés de resposta, em que os profissionais de saúde podem ter relatado o que acreditavam ser a prática correta em vez do seu comportamento de rotina real. Em todo o caso, tratando-se de números auto-reportados, estão sem dúvida sujeitos a um viés de memória.

Surpreendentemente, 59% dos profissionais no nosso estudo tinham ouvido falar do noma, o que é comparável aos participantes no estudo zambiano[11] em torno de 54%, mas muito inferior à taxa de 91% relatada no Burkina Faso,[12] ou aos 78,8% a 83,7% no Noroeste da Nigéria.[14,15] No entanto, 34% dos participantes no Burkina Faso tinham participado anteriormente numa formação sobre o noma; e, por conseguinte, a percentagem na comunidade de saúde em geral pode ser inferior. Pelo contrário, no Noroeste da Nigéria, os estudos foram realizados a cinco quilómetros de distância do Hospital Pediátrico de Noma, onde muitas campanhas de sensibilização e investigação têm realizadas há várias décadas,[13] e, portanto, seria de esperar um nível de conhecimento mais elevado do que em Moçambique, onde, tanto quanto sabemos, ainda não foi realizada qualquer formação específica sobre o noma.

Apesar de a maioria dos participantes já ter ouvido falar da doença, quando lhes foram apresentados casos práticos de noma, apenas 12% dos participantes conseguiram diagnosticar corretamente o noma no estádio 1 (ANUG) e 5% no estádio 2 (edema), que ainda só são reversíveis se for feito um diagnóstico e administrado o tratamento adequado. Mais participantes (46%) conseguiram diagnosticar o noma na fase 4 (cicatrização), 79% entre os que já tinham ouvido falar da doença anteriormente. Na Zâmbia, todos os participantes tinham um nível de escolaridade pré-universitário e os seus resultados foram comparáveis aos da nossa subamostra moçambicana com o mesmo nível de escolaridade.[11] Embora os participantes zambianos tenham demonstrado uma maior taxa de acerto no diagnóstico do estádio 1 (71%), isto deveu-se em grande parte à sua inclusão de gengivite simples como resposta correta, representando 69% das respostas.[11] Utilizando a definição da OMS para o estádio 1,[2] no entanto, os participantes moçambicanos superaram o grupo zambiano (7% vs 2% de acerto). Para os casos de estádio 2 e estádio 4, a correção do diagnóstico foi igualmente avaliada em ambos os estudos e os resultados foram comparáveis (6% no estádio 2 para os participantes zambianos versus 3% no nosso estudo, e 29% no estádio 4 na Zâmbia em

comparação com 34% em Moçambique). A correção muito baixa do diagnóstico para o estádio 2 pode ser parcialmente explicada pelo facto de os sinais e sintomas descritos no caso prático não serem exclusivos do noma.

Apesar de terem errado no diagnóstico, cerca de 60% dos participantes recomendaram a amoxicilina para o tratamento das fases 1 e 2, mas menos de 20% mencionaram o metronidazol e a gentamicina, o que é consistente com os resultados do estudo da Zâmbia.[11] O apoio nutricional e a reidratação, aspectos críticos dos cuidados com o noma, foram os tratamentos mais frequentemente negligenciados, juntamente com a referenciação, que se torna crucial a partir da fase 2 devido à rápida progressão da doença para necrose. Apenas 24% dos profissionais teriam encaminhado uma criança com fase de edema para uma unidade de saúde de nível superior. Na fase tardia da cicatrização, quando a fase aguda da doença já está concluída, 52% dos participantes de nível pré-universitário aconselharam o encaminhamento, o que está logo abaixo das taxas de encaminhamento de 70 a 100% relatadas por enfermeiros no Burkina Faso.[12] Entre os participantes de nível pré-universitário, 52% recomendaram o encaminhamento na Zambézia, em comparação com a taxa de encaminhamento de 31% relatada pelo estudo zambiano.[11] A competência total de gestão foi considerada "muito baixa" (<25%) para 72% dos participantes do estudo pré-universitário, o que contrasta com a pontuação "subóptima" (25-49%) da maioria dos enfermeiros participantes do Burkina Faso, tendo apenas 8% uma pontuação "muito baixa".[12] No entanto, o seu sistema de pontuação não está claramente detalhado e a diferença pode dever-se a um sistema de pontuação mais laxista que conduz a uma aparente melhor competência de gestão

O conhecimento teórico sobre a doença entre os participantes que já tinham ouvido falar sobre o noma foi aparentemente melhor do que a competência de gestão, no entanto, isto pode dever-se parcialmente ao facto de ter sido avaliado por perguntas fechadas com respostas disponíveis, ao contrário da competência de gestão que foi avaliada por perguntas

abertas. No estudo da Zâmbia, os conhecimentos teóricos foram avaliados através de perguntas abertas e o nível de conhecimentos gerais foi classificado como "muito baixo" em 66% dos participantes,[11] contrastando com 55% dos enfermeiros do Burkina Faso[12] e 93% dos profissionais pré-universitários em Moçambique que obtiveram uma pontuação "boa" ou "óptima". É de salientar que, apesar de Sokoto ser um epicentro de investigação e tratamento do noma há décadas, os profissionais de saúde da Zambézia superaram os seus homólogos de Sokoto em determinadas questões. Em particular, menos participantes em Sokoto identificaram a má higiene oral como um fator de risco para o noma (68,3% vs 100%), ou reconheceram a utilização de antibióticos como tratamento (37,4% vs 96%).[14] Estudos futuros em ambas as regiões devem abordar estas variações e esforçar-se por gerar provas mais robustas e comparáveis.

Notavelmente, 26,8% dos participantes relataram ter assistido pessoalmente a um doente com noma agudo durante as suas carreiras, um número que contrasta com os 7% relatados no Burkina Faso,[12] e os 11% na Zâmbia.[11] Uma possível explicação para esta diferença é que quase metade dos que relataram ter assistido a um doente agudo no nosso estudo eram médicos ou estomatologistas, enquanto os estudos do Burkina Faso e da Zâmbia incluíram principalmente enfermeiros e profissionais de saúde, respetivamente. A nossa hipótese é que os médicos têm maior probabilidade de ter trabalhado em hospitais de referência que cobrem grandes áreas de captação, para onde são transferidas as doenças graves.

Em Moçambique, o primeiro ponto de entrada para os cuidados de saúde para a maioria da população rural são os curandeiros tradicionais, agentes comunitários de saúde (*agentes polivalentes*), ou técnicos médicos nos centros de cuidados primários. No entanto, e como evidenciado pelo seu melhor desempenho neste estudo, a formação do noma tem atualmente como alvo os especialistas em saúde oral, cujo número é criticamente baixo. Alguns técnicos de saúde, enfermeiros e médicos tinham ouvido falar do noma durante os seus estudos,

apesar de não estar oficialmente incluído nos seus currículos, no entanto, a sua competência de gestão era muito baixa. Para melhorar a deteção precoce e a gestão, os curandeiros tradicionais e os trabalhadores dos cuidados primários devem receber formação para reconhecer os sinais precoces e encaminhar os doentes quando necessário. Para além disso, os médicos, pediatras, dentistas e nutricionistas devem saber como gerir os casos agudos, enquanto os fisioterapeutas, psicólogos e cirurgiões maxilofaciais e plásticos são essenciais para tratar as sequelas funcionais e psicológicas do noma a longo prazo

Como observado no nosso estudo, e corroborado noutros países, os curandeiros tradicionais,[16,17] e os trabalhadores dos cuidados primários expressam vontade de receber formação sobre o noma[12,15]. Mensagens chave podem ser transmitidas efetivamente através de programas de formação curtos para enfermeiros,[12] e para curandeiros tradicionais, [27] como demonstrado no Burkina Faso. A implementação de tais programas não é apenas imperativa do ponto de vista da garantia do direito humano à saúde,[28] mas também para evitar os custos indirectos das mortes prematuras, que ascendem a milhares de milhões a nível nacional.[29]

A principal limitação deste estudo reside na sua representatividade restrita. Devido a restrições de recursos, a amostragem foi efectuada por conveniência e o tamanho da amostra foi pequeno. Além disso, a maioria das unidades de saúde visitadas situava-se perto de zonas urbanas, o que pode não refletir com exatidão as zonas rurais. Por último, não incluímos agentes comunitários de saúde nem curandeiros tradicionais, ambos cruciais para o diagnóstico precoce do noma. Além disso, embora a equipa tenha tido o cuidado de evitar qualquer menção ao noma antes do questionário, os participantes podem ter-se apercebido previamente do tópico do estudo, podendo assim influenciar as respostas de diagnóstico na secção de competências de gestão. Outra limitação é que não avaliámos a competência de gestão para o estadio 3 do noma, e o caso prático utilizado para avaliar o estadio 2 do noma não era suficientemente específico, o que dificultou a fiabilidade dos resultados. Por fim, os

dados sobre o número de pacientes atendidos estão sujeitos a viés de memória, e devem ser contrastados com dados de saúde de rotina em estudos futuros.

Conclusões

A prevalência do noma em Moçambique permanece indeterminada; no entanto, 56% dos participantes neste estudo relataram ter encontrado um doente com noma na sua prática ou na sua vida pessoal. O tratamento do noma estava prontamente disponível nas unidades sanitárias visitadas, bem como a possibilidade de encaminhamento. No entanto, a maioria dos profissionais de saúde não conseguia identificar os estágios iniciais do noma, nem conhecia sua alta mortalidade, sua rápida progressão ou seu manejo correto. Assim, não se trata de falta de acesso ao tratamento, mas de falta de conhecimento sobre a doença, o que dificulta o diagnóstico atempado e os cuidados adequados. É urgente implementar programas de formação abrangentes a todos os níveis de prestadores de cuidados de saúde, para garantir o direito fundamental à saúde. Estudos de investigação de implementação que incorporem as perspectivas dos sobreviventes de noma e das suas comunidades serão fundamentais para identificar as estratégias mais eficazes de formação e sensibilização nos contextos socioculturais e de cuidados de saúde locais.

Agradecimentos

Gostaríamos de agradecer aos profissionais de saúde que dedicaram tempo para responder às nossas perguntas e aprender sobre o noma. Agradecemos a Elise Farley, que gentilmente reveu o nosso questionário. Gostaríamos também de agradecer aos directores dos hospitais visitados que permitiram que o trabalho se realizasse com sucesso. Finalmente, gostaríamos de agradecer a todos os contribuintes para o crowdfunding que financiou parcialmente este projeto.

Declaração de informação financeira

Este estudo foi financiado pela ISGlobal e pela Universidade de Navarra através de uma campanha de crowdfunding. MR foi apoiado por uma bolsa de doutoramento AGAUR-FI_B 01022 do Governo Catalão e pelo Fundo Social Europeu. GM foi apoiado pelo RYC2020-029886-I/AEI/10.13039/501100011033, cofinanciado pelo Fundo Social Europeu (FSE). Reconhecemos o apoio à ISGlobal através da subvenção CEX2023-0001290-S financiada pelo MCIN/AEI/10.13039/501100011033, e o apoio da Generalitat de Catalunya através do Programa CERCA.

Os financiadores não tiveram qualquer papel na conceção do estudo, na recolha e análise de dados, na decisão de publicar ou na preparação do manuscrito.

Contribuições do autor

Conceptualização: MR, FP, CCh

Metodologia: MR, AA, FP, CCh

Análise formal: MR, FH, GM, CCh

Investigação: AA, EE, MA, MR, TS, LT, FP

Curadoria de dados: RR, MR, FH

Visualização: EE, MR

Redação - projeto original: MR

Redação - revisão e edição: todos os autores contribuíram, reveram e aprovaram a versão final do manuscrito

Supervisão: GM, FP, CCh

Aquisição de financiamento: CCh, MR

Declaração de interesses

Não declaramos quaisquer interesses concorrentes.

Declaração de partilha de dados

Os dados individuais dos participantes, anónimos, estão disponíveis em https://github.com/marta-ribes/Noma-Echoes-KAP-questionnaire.git. Uma versão deste artigo em português, bem como o instrumento de recolha de dados, estão disponíveis como Material Suplementar.

A assistência do ChatGPT da OpenAI foi utilizada para melhorar a legibilidade e a correção gramatical durante a preparação deste manuscrito. Após a utilização desta ferramenta, os autores reviram e editaram o conteúdo conforme necessário e assumem total responsabilidade pelo conteúdo da publicação.

Referências

1. Farley E, Mehta U, Leila Srour M, Lenglet A. Noma (cancrum oris): Uma revisão da literatura de escopo de uma doença negligenciada (1843 a 2021). PLoS Negl Trop Dis [Internet]. 2021 Dez 1 [citado 2022 Jul 21];15(12). Disponível em: /pmc/articles/PMC8670680/

2. Gabinete Regional da OMS para África. Brochura informativa para a deteção precoce e a gestão do noma [Internet]. 2016. Disponível em: www.panacee.fr

3. Kagoné M, Mpinga EK, Dupuis M, Moussa-Pham MSA, Srour ML, Grema MSM, et al. Noma: Experiences of Survivors, Opinion Leaders and Healthcare Professionals in Burkina Faso (Experiências de Sobreviventes, Formadores de Opinião e Profissionais de Saúde no Burkina Faso). Trop Med Infect Dis. 2022 Jul 1;7(7).

4. Uzochukwu I, Moyes D, Proctor G, Ide M. Os principais actores da disbiose na doença de Noma; Uma revisão sistemática dos estudos etiológicos. Vol. 4, Frontiers in Oral Health. Frontiers Media S.A.; 2023.

5. Huyghe A, François P, Mombelli A, Tangomo M, Girard M, Baratti-Mayer D, et al. Microarray Analysis of Microbiota of Gingival Lesions in Noma Patients. PLoS Negl Trop Dis [Internet]. 2013 [citado 2024 Mar 6];7(9):e2453. Disponível em: https://journals.plos.org/plosntds/article?id=10.1371/journal.pntd.0002453

6. Galli A, Brugger C, Fürst T, Monnier N, Winkler MS, Steinmann P, et al. Revisão Prevalência, incidência e distribuição global relatada de noma: uma revisão sistemática da literatura. 2022 [citado 2022 Ago 17]; Disponível em: www.thelancet.com/infection

7. Srour ML, Farley E, Mpinga EK. Sobreviventes de Noma do Laos: Uma série de casos, 2002-2020. A Sociedade Americana de Medicina Tropical e Higiene. 2022;106(4):1269-74.

8. Montandon D. Cirurgia do noma: uma experiência de 20 anos. Stomatologie. 2007 Mar;104(1):1-9.

9. Equipa de Comunicação Social da Organização Mundial de Saúde (OMS). OMS reconhece oficialmente o noma como uma doença tropical negligenciada [Internet]. 2023 [citado 2024 Jul 30]. Disponível em:

https://who.int/news/item/15-12-2023-who-officially-recognizes-noma-as-a-neglected-tropical-disease

10. The Lancet Global Health. Noma: já não é negligenciado? Lancet Glob Health. 2024 Feb 1;12(2):e170.

11. Ahlgren M, Funk T, Marimo C, Ndiaye C, Alfvén T. Management of noma: Practice competence and knowledge among healthcare workers in a rural district of Zambia. Glob Health Action [Internet]. 2017 [citado 2023 Fev 6];10(1). Available from: https://www.tandfonline.com/doi/abs/10.1080/16549716.2017.1340253

12. Brattström-Stolt L, Funk T, Sié A, Ndiaye C, Alfvén T. Noma-knowledge and practice competence among primary healthcare workers: a cross-sectional study in Burkina Faso. Int Health [Internet]. 2019 Jul 1 [citado 2024 Abr 11];11(4):290-6. Disponível em: https://dx.doi.org/10.1093/inthealth/ihy088

13. Isah S, Amirtharajah M, Farley E, Semiyu Adetunji A, Samuel J, Oluyide B, et al. Modelo de cuidados, Noma Children's Hospital, noroeste da Nigéria. Medicina Tropical e Saúde Internacional. 2021 Sep 1;26(9):1088-97.

14. Mujtaba B, Chimezie CB, Braimah RO, Taiwo AO, Adebayo IA, Ndubuizu GU, et al. Knowledge, Attitude and Practices of Health Care Workers towards NOMA in a Tertiary Institution in North-western Nigeria. Jornal Nigeriano de Investigação Dentária. 2022 Aug 10;7(2):110-5.

15. Bala M, Omotayo SA, Braimah RO, Taiwo AO, Jaafar R, Abubakar AB, et al. Knowledge, Attitude, and Practices of Primary Health-Care Workers toward NOMA Disease in Sokoto. Dentistry and Medical Research. 2022 Jan;10(1):24-8.

16. Farley E, Bala HM, Lenglet A, Mehta U, Abubakar N, Samuel J, et al. 'I treat it but I don't know what this disease is': a qualitative study on noma (cancrum oris) and traditional healing in northwest Nigeria. Int Health [Internet]. 2020 Jan 1 [citado 2024 Abr 11];12(1):28-35. Disponível em: https://dx.doi.org/10.1093/inthealth/ihz066

17. Baratti-Mayer D, Daou MB, Gayet-Ageron A, Jeannot E, Pittet-Cuénod B. Sociodemographic Characteristics of Traditional Healers and Their Knowledge of Noma: Um inquérito descritivo em três regiões do Mali. Revista Internacional de Investigação Ambiental e Saúde Pública 2019, Vol 16, Página 4587 [Internet]. 2019 Nov 19 [citado 2023 Feb 6];16(22):4587. Available from: https://www.mdpi.com/1660-4601/16/22/4587/htm

18. Fernandes Q, Augusto O, Machai H, Pfeiffer J, Carone M, Pinto N, et al. Escrutínio da disponibilidade e distribuição de recursos humanos para a saúde em Moçambique entre 2016 e 2020: um estudo longitudinal descritivo subnacional. Hum Resour Health. 2023 Dez 1;21(1).

19. Manaca MN. RELATÓRIO FINAL AVALIAÇÃO RÁPIDA DA SITUAÇÃO DE NOMA EM MOÇAMBIQUE [Internet]. 2023 Jun [cited 2024 Jul 30]. Disponível em: https://www.misau.gov.mz/boletins-epidemiologico/

20. Palmira Fortunato dos Santos. Curandeiros modernos e tradicionais unem forças para melhorar os cuidados com a epilepsia em Moçambique. Liga Internacional Contra a Epilepsia [Internet]. 2019 [cited 2024 Oct 1];21(4). Disponível em: https://www.ilae.org/journals/epigraph/epigraph-vol-21-issue-4-fall-2019/modern-and-traditional-healers-combine-forces-to-improve-epilepsy-care-in-mozambique

21. Instituto Nacional de Estatística. POPULACAO POR IDADE- SEGUNDO AREA DE RESIDENCIA- NACIONALIDADE E SEXO. PROVINCIA DA ZAMBEZIA. https://ine.gov.mz/web/guest/d/quadro-4-populacao-por-idade-segundo-area-de-residencia-nacionalidade-e-sexo-provincia-da-zambezia-2017. 2017.

22. OMS. Portal de dados das contas nacionais da mão de obra no sector da saúde [Internet]. 2023. 2024 [citado 2024 Out 5]. Disponível em: https://apps.who.int/nhwaportal/

23. Força de trabalho no sector da saúde. Requisitos da força de trabalho em saúde para a cobertura universal de saúde e os Objectivos de Desenvolvimento Sustentável [Internet]. Genebra; 2016 [citado 2024 Out 13]. Disponível em: https://www.who.int/publications/i/item/9789241511407

24. Harris PA, Taylor R, Minor BL, Elliott V, Fernandez M, O'Neal L, et al. The REDCap consortium: Building an international community of software platform partners. J Biomed Inform. 2019 Jul;95:103208.

25. Harris PA, Taylor R, Thielke R, Payne J, Gonzalez N, Conde JG. Research electronic data capture (REDCap) - Uma metodologia orientada por metadados e um processo de fluxo de trabalho para fornecer apoio informático à investigação translacional. J Biomed Inform. 2009 Apr;42(2):377-81.

26. Domingos MAAM, Mepatia AI, Xavier CNH, Barrie RB, Naidoo S, Marsicano JA, et al. Cárie dentária e doenças periodontais em Moçambique. Investigação, Sociedade e Desenvolvimento. 2022 Jan 12;11(1):e46511125221.

27. Baratti-Mayer D, Jeannot E, Dupuis M. Implementation and Evaluation of a Training Program for Traditional Healers to Improve Knowledge of Noma (Cancrum Oris) in Burkina Faso. Am J Trop Med Hyg. 2024 Feb 7;110(2):303-10.

28. Nações Unidas. Human rights bodies and mechanisms Study of the Human Rights Council Advisory Committee on severe malnutrition and childhood diseases with children affected by noma as an example. In: Estudo do Comité Consultivo do Conselho dos Direitos Humanos sobre a desnutrição grave e as doenças infantis tendo como exemplo as crianças afectadas pelo noma. 2012. p. 1-22.

29. Mpinga EK, Srour ML, Moussa MSA, Dupuis M, Kagoné M, Grema MSM, et al. Economic and Social Costs of Noma: Design and Application of an Estimation Model to Niger and Burkina Faso. Trop Med Infect Dis. 2022 Jul 1;7(7).
