# Supplementary material for: Knowledge, attitudes and practices of healthcare workers towards noma in Zambezia, Mozambique
Source: PLoS Negl Trop Dis. 2025 Mar 19;19(3):e0012939. doi: 10.1371/journal.pntd.0012939 (PMC11952756; doi:10.1371/journal.pntd.0012939)
Supplement: Attachment — Submitted filename: 20250201 answers to reviewers PNTD-D-24-01714.pdf [file pntd.0012939.s004.pdf]

Reviewer #1 comments:

Reviewer comment	Answer to comment	Change in manuscript
The conclusion section needs to be shortened and focused on the key insights from the study.	Thank you for this suggestion	We have moved some argumentations to the discussion section. See track changes.
- line 39 “dreadful”, line 79 “outrageous” – avoid emotional language here and throughout the manuscript	Thank you for your suggestion.	We have changed outrageous for “striking”, and dreadful for “extremely”
- line 59-60, 494-495: rather than “lack of access to timely and appropriate care” the issue could be called more simply “lack of diagnostic capacity” (or similar)	Thank you. We would like to still reflect that time is also crucial.	We have changed to “lack of awareness about the disease, which hinders timely diagnosis and appropriate care”
- line 81, line 130: “easily”, “brief” – without context it is difficult to judge what the authors claim was “easy” identification of noma cases as a short duration alone can not indicate how easy something was	Understood.	We have suppressed “easily” and “brief”
- line 111-112: 70% had sub-optimal competence and half had good knowledge which adds up to over 100%	The study measured practice competences and theoretical knowledge separately, so percentages correspond to two separate proportions.	None
- line 119-120: the sentence should be deleted as it is offering a criticism without argument to support it	Agreed.	This has been done
- line 126-127: this is unclear as traditional healers are said to recognize treating noma but at the same time not knowing which disease it was	Thank you	We have clarified it changing it for “attending noma patients”
- line 147: how was the study province selected?	Thank you for alerting us of this missing information.	We have added: “The study site was chosen based on

		prior informal reports of noma patients living in the province.”
- line 125-129: these numbers appear to be self-reported by the respondents. Or were medical records reviewed? Please clarify and discuss the option of record review in the discussion section.	We assume you are referring to lines 225 to 229.	We have clarified it is self-reported and included the potential recall bias in the limitations.
- line 400-404: consider integrating this into another paragraph or deleting it as the content is not warranting a separate sub-chapter and is not relevant for the rest of the study	Thank you	We have removed the sub-title and integrated the text into the section above
- line 406: “quarterly” – do the authors mean quaternary?	Yes.	Changed accordingly.
- Conclusions section: this should be more concise, focusing only on the key messages derived from the study	Agreed.	We shortened this section accordingly. See track changes.
- the authors are invited to discuss in more detail the relationship between the quality of answers / correctness of diagnoses based on the vignettes and the reported awareness of the disease / previous training	Given the small sample size, we cannot compute correlations between these variables. However, we have provided additional points about them in the discussion.	See additional arguments in lines 473 and 537.
- it seems that noma was part of the training curriculum of many health professionals. Do the authors have information which courses specifically? Also, it is claimed that generally, noma were not part of training (line 103) but the reported figures suggest otherwise.	Officially, it is only included in the curricula of dentists and maxillofacial surgeons.	It was included in the introduction, and is further discussed now in the discussion.
- it should be discussed that while knowledge about noma, especially its early signs, was higher among well-trained staff, it is most urgently needed in peripheral facilities where affected populations seek help first	Agreed.	Discussed in the discussion (page 26).

Reviewer #2 comments:

Reviewer comment	Answer to comment	Change in manuscript

However, the objectives of the study are not clearly outlined in the Methods section. I suggest incorporating them at the beginning of the "Procedures" paragraph (end of line 163). Alternatively, consider presenting them more comprehensively at the end of the Introduction (lines 144–145). Currently, only the main objective (knowledge on noma) is mentioned (lines 144-145), while the secondary objectives (assessment of practices related to oral health and attitudes toward noma training) are not addressed.	Thank you for this suggestion of improvement.	We have added to line 145 “, focusing on both theoretical understanding and practical management competencies.”
In some sections, the data presentation is overly detailed. To make the text more concise and easier to read, the authors could consider omitting some of the data described in the Results, directing readers to the tables for additional details—particularly in the subsection “Management competences”.	Thank you for your suggestions. We agree, however, some aspects do warrant highlighting in the narrative to emphasize central concepts	We have sought a balance and the text has been shortened in response to other reviewers which may ease the general narrative.
I suggest moving lines 232–241 to the subsection titled “Specific knowledge on noma,” placing them after line 354 (“...21% at work and one during a course on HIV”). This adjustment would allow the authors to first report how many professionals are aware of noma and then follow with how many have actually encountered cases.	Thank you for your suggestion.	We have moved this section to the end of the “Theoretical knowledge” section.
Lines 236–241 seem to add limited value to the subject due to their anecdotal nature. I suggest deleting these lines and simply stating that the professionals who encountered noma cases were from different geographical areas, with the number of cases per professional ranging from 1 to 12.	Given how little information there is on noma, we consider these are still interesting and prefer to leave it in the manuscript.	No action.
I believe the need to train healthcare personnel on noma is a crucial aspect of the article, given its significant practical implications for public health. However, this topic is mentioned only in the Conclusions rather than being explored in the Discussion. Thus, I suggest revising as follows:<br><br>- Move lines 496–502 (“In Mozambique, the first entry point… as demonstrated in Burkina Faso”) to the end of the Discussion (after line 485). Follow this by adding lines 504–508 (“Traditional healers and primary care workers… and long-term sequelae”). Consider expanding the discussion on the topic with practical examples	Thank you for this suggestion. It will improve readability.	Suggestion incorporated.

of how training has been implemented in other settings or by referencing experiences beyond Burkina Faso.		
Keep lines 503–504 and 508–509 in the Conclusions (following line 495): "It is therefore not a matter of lack of access to treatment, but lack of access to timely and appropriate care. It is crucial to implement comprehensive training programs across all levels of healthcare providers. Implementation research studies which incorporate the perspectives of noma survivors and their communities, will be key to identify the most effective strategies for training and awareness raising within local healthcare and sociocultural contexts." Consider adding further details on why educating healthcare personnel on noma is so important by highlighting economic and social implications (eg., preventing disabilities and psychological impacts on children; avoiding the need for complex reconstructive surgeries).	This is a great suggestion. Thank you.	Suggestion incorporated.
Line 31: spelling mistake "sixty-three" inestead of "tree"	Thank you	Changed accordingly
Line 176-7, "…Médecins Sans Frontières in Sokoto State, Nigeria" there's a missing quote	The article is not published yet, this was a personal communication.	This personal communication has been cited appropriately
Line 222: as stated in Table 1, authors should replace "34" with "24": "11% 16 to 24 and 22% over 24"	Thank you	Changed accordingly
Line 223: I suggest adding "attended", instead of "tended to": "…university-level professionals attended fewer patients"	Thank you	Changed accordingly
Line 324: According to Table 2, "99% vs 48% p<0.05" should be "9% vs 48% p <0.05"	Thank you	Changed accordingly
Line 333: The authors should provide a definition for the acronym "CHW," as it is not previously mentioned in the paper.	Thank you	Changed accordingly
Line 359: The acronym ANUG, referring to the first stage of noma, is here used without explanation. Since the full term is firstly mentioned at line 85, I suggest providing a definition for the acronym there. Additionally, the acronym ANUG should be consistently used in lines 266, 359, and 437.	Thank you	Changed accordingly
Lines 440-2: add reference 11	Thank you	Changed accordingly
Line 445: add reference 2 for WHO definition	Thank you	Changed accordingly
Line 406: I would change "quaterly" to "quaternary"	Thank you	Changed accordingly

Line 494: I think “access to treatment” and “access to appropriate care” are nearly synonyms; I would rather stress the existing challenges in identifying the disease and taking action to treat it effectively. I would change line 494 to: “It is therefore not a matter of lack of access to treatment, but lack of awareness about the disease, which hinders timely diagnosis and appropriate care”	Thank you for this useful suggestion.	Changed accordingly
Table 3, table header: I would change “mean” to “mean/%” and “median” to “median/%”, in line with Table 2.	Thank you	Changed accordingly
Table 3, 6th row: authors should clarify why only 21 participants, rather than 24, responded to the question "Can you order noma stages?"	Thank you for alerting us, this was our mistake. Three participants said “did not know” and we had mistakenly not included them in the analysis.	Proportions and statistics updated, the estimate and p-value have varied very little.
